# Zoonotic potential of *Enterocytozoon bieneusi* among children in rural communities in Thailand

**DOI:** 10.1051/parasite/2013014

**Published:** 2013-04-11

**Authors:** Hirotake Mori, Aongart Mahittikorn, Dorn Watthanakulpanich, Chalit Komalamisra, Yaowalark Sukthana

**Affiliations:** 1 Department of Protozoology, Faculty of Tropical Medicine, Mahidol University Bangkok Thailand; 2 Department of Helminthology, Faculty of Tropical Medicine, Mahidol University Bangkok Thailand

**Keywords:** microsporidia, zoonosis, *Enterocytozoon bieneusi*, children, Thailand

## Abstract

*Enterocytozoon bieneusi* is a common opportunistic intestinal pathogen worldwide. Genotype distribution of *E. bieneusi* differs by geography and host immunity. In order to investigate the prevalence, genotype characteristics, and host specificity of *E. bieneusi* in the community, we conducted a preliminary cross-sectional study among children in Western and Northern Thailand. Seventy-eight (78) and 102 stool samples were collected; the prevalence of *E. bieneusi* was 3.8% and 2.9% by nested PCR in Western and Northern Thailand, respectively. Three genotypes were identified: Genotype D predominated, followed by EbpC, and then novel genotype ETMK1. The first two genotypes have zoonotic potential. Analysis of the genetic proximity of the *E. bieneusi* ITS sequences from our study, compared with those published in genetic databases, showed that all positive samples were classified into Group 1, the largest group consisting of various host specificity. The present study demonstrates the possible zoonotic transmission of *E. bieneusi* in rural communities in Thailand. A large-scale investigation of both human and animal samples, as well as improvements in the available phylogenetic tools, will be required to elucidate transmission routes of *E. bieneusi* in this area.

## Abstract (fr)

*Enterocytozoon bieneusi* est un pathogène intestinal opportuniste commun et mondial. La distribution des génotypes d’*E. bieneusi* change selon la géographie et l’immunité des hôtes. Pour étudier la prévalence, les caractéristiques des génotypes et la spécificité aux hôtes d’*E. bieneusi* dans la communauté, nous avons effectué une étude transversale préliminaire chez des enfants de l’ouest et du nord de la Thaïlande. Soixante-dix-huit (78) et 102 échantillons de selles ont été récoltés. La prévalence d’*E. bieneusi*, étudiée par PCR, était de 3,8 % et 2,9 %, respectivement, dans l’ouest et le nord de la Thaïlande. Trois génotypes ont été identifiés : le génotype D prédominait, suivi par EbpC, et par le génotype nouveau ETMK1. Les deux premiers génotypes ont un potentiel zoonotique. L’analyse de la proximité génétique des séquences ITS des *E. bieneusi* de notre étude, comparées avec celles publiées dans les bases de données, montre que tous les échantillons positifs sont classés dans le Groupe 1, le plus grand groupe, qui inclut des spécificités d’hôtes variées. Cette étude démontre la possible transmission zoonotique d’*E. bieneusi* dans les communautés rurales de Thaïlande. Une étude à grande échelle d’échantillons à la fois humains et animaux et des améliorations dans les outils phylogénétiques disponibles seront nécessaires pour élucider les voies de transmission d’*E. bieneusi* dans cette zone.

## Introduction

*Enterocytozoon bieneusi* Desportes et al., 1985 [8] is the most common cause of intestinal microsporidiosis. *E. bieneusi* infections in immunocompetent hosts are usually self-limiting, while infections in immunocompromised hosts can be life-threatening, especially in patients with AIDS [8]. Recently, awareness of microsporidiosis in non-HIV-infected populations has increased, and infections among organ-transplant recipients, children, the elderly, and patients with malignant disease and diabetes, have been reported [10]. Microscopic diagnosis of *E. bieneusi* is difficult because the organism is small and similar in size to bacteria. Molecular techniques, such as PCR, are more sensitive than microscopy and have been more widely used in recent times [14–16]. The numbers of *E. bieneusi* genotypes, based on the internal transcribed spacer (ITS) nucleotide sequence of the ribosomal RNA gene, have increased rapidly; currently, over 100 genotypes have been published in GenBank [29, 30].

The genotype distribution of *E. bieneusi* differs by geography. Anthroponotic host-specific genotypes are frequently observed in developed countries, while in developing areas, both host-specific and non-host-specific genotypes have been identified [4, 7, 33]. In addition, recent molecular epidemiological studies have demonstrated that genotype distribution differs between HIV and non-HIV patients [23, 36]. In Thailand, zoonotic genotype D is most commonly identified in HIV patients, however, only anthroponotic genotype A has been identified in the community and in non-HIV individuals [20–22]. Factors influencing this difference in genotype distribution are not clearly understood. There may be an important association in the host/organism relationship, such as host immune status, virulence, or host specificity of the organism itself [23]. So far, molecular epidemiological studies of *E. bieneusi* have been mainly conducted in HIV and non-HIV patients, while only a few studies have been conducted in the community. For a better understanding of the basic epidemiological characteristics of the organism, such as infection sources and zoonotic potential, surveillance in rural communities is required.

Therefore, we conducted a cross-sectional study among children in rural communities in Western and Northern Thailand. We investigated the prevalence of *E. bieneusi* by nested PCR, genotype characteristics, and host specificity. A phylogenetic tree was constructed for further evaluation of zoonotic potential.

## Materials and methods

### Study design

In Thailand, in June and December 2011, 79 and 102 stool samples were collected from children in Kanchanaburi (age 4–12 years) and Nan (age 4–6 years) Provinces respectively. The community in Kanchanaburi Province is located on the Thai-Myanmar border, in Western Thailand. The village in Nan Province is located on the Thai-Lao border, in Northern Thailand. Both communities are known endemic areas for parasitic infections, due to low socio-economic status and poor hygiene standards. The parents of the children received instructions for stool collection and provided consent for the investigation. Stool samples were kept in cool conditions during transportation and preserved at −80 °C until DNA extraction.

### DNA extraction, PCR amplification, and nucleotide sequencing

DNA was extracted from the samples using a commercially available DNA extraction kit (PSP Spin Stool DNA Kit, STRATEC Inc., Germany) in accordance with the manufacturer’s instructions. Acquired DNA was stored at −20 °C. A nested PCR was performed to amplify a fragment of the large and small subunit of the rRNA gene, including the entire ITS region. The outer primer pair was EBITS3 (5′-GGT CAT AGG GAT GAA GAG-3′) and EBITS4 (5′-TTC GAG TTC TTT CGC GCT C-3′). The inner primer pair was EBITS1 (5′-GCT CTG AAT ATC TAT GGC T-3′) and EBITS2.4 (5′-ATC GCC GAC GGA TCA AGT G-3′) [5]. Each 25 μl PCR mixture contained 1× PCR buffer, 1.5 mM MgCl_2_, 0.2 mM dNTPs, 2.5 U Taq polymerase (Fermentas, USA), and 0.25 μM of each primer. For primary and secondary PCR, reaction conditions were designed as follows: 35 cycles of 94 °C for 1 min, 55 °C for 1 min, and 72 °C for 1 min. Two microliters of the initial PCR products was used as the template for secondary PCR. Secondary PCR produced fragments of 390 bp. The PCR products were subjected to electrophoresis in a 2% agarose gel and visualized by staining the gel with ethidium bromide. All amplified products were sequenced in both directions using the secondary PCR primers EBITS1 and EBITS2.4 on an ABI 3730xl DNA analyzer (Applied Biosystems). The genotypes of *E. bieneusi* from each specimen were confirmed by the homology of the sequenced PCR products to the published sequence in GenBank.

### Genetic proximity of the *E. bieneusi* ITS sequences

Analysis of the genetic proximity of the *E. bieneusi* ITS sequences from different origins was performed using MEGA Software Version 4 [35]. The evolutionary distance between the different isolates was calculated using the Kimura 2-parameter method, and phylogenetic trees were constructed using the neighbor-joining algorithm. Branch reliability was assessed using bootstrap analyses (1000 replicates).

### Ethical approval

This study was approved by the Ethics Committee of the Faculty of Tropical Medicine, Mahidol University (MUTM 2012-064-01).

### Nucleotide sequence accession numbers

The sequence of *E. bieneusi* novel genotype ETMK1 in the present study was submitted and deposited in GenBank with Accession No. JX914568.

## Results

Prevalence and genotypes of *E. bieneusi* positive samples are shown in Table 1. The prevalence of *E. bieneusi* was 3.8% and 2.9% in Kanchanaburi and Nan Provinces, respectively. In Kanchanaburi province, three samples (3.8%) were positive; two samples were genotype D and one was novel genotype ETMK1. ETMK1 was one base different from genotype EbfelA, L and V as shown in Table 2 (EbfelA position 93 [A→C]; L position 118 [A→G]; V position 130 [G→A]). In Nan Province, three samples (2.9%) were positive. Two samples were genotype D and one was genotype EbpC.

**Table 1.** Prevalence and genotypes of *E. bieneusi* among children in Kanchanaburi and Nan provinces, Thailand.

Source (Province)	Number of samples examined	Number of positive samples (Prevalence %)	Genotype (number of samples)	Subtype
Kanchanaburi	78	3 (3.8%)	D (2)	1a
			ETMK1 (1)	1a
Nan	102	3 (2.9%)	D (2)	1a
			EbpC (1)	1d
Total	180	6 (3.3%)

**Table 2.** Polymorphic sites in ITS sequences of *E. bieneusi* isolates.

Position No.	31	77	93	113	117	118	130	137	141
D	G	G	C	C	T	G	G	C	T
V	–	–	A	–	–	A	A	–	–
ETMK1	–	–	A	–	–	A	–	–	–
L	–	–	A	–	–	–	–	–	–
EBfelA	–	–	–	–	–	A	–	–	–
A	–	–	T	–	G	–	–	T	–
B	A	A	T	–	G	–	–	T	–
K	–	–	T	–	G	–	A	–	–
EbpC	–	–	T	A	G	–	–	–	C

The result of the phylogenetic tree was poorly reliable due to low bootstrap values (<20) in the internal branches. However, the majority of the classification in the phylogenetic tree matched with the study by Thellier and Breten [37]; therefore, we followed their classification. Group 1, the largest clade, is further subdivided into eight clades (subgroups 1a–1h). All of the positive samples in the present study were classified into Group 1; genotype D and ETMK1 were classified into subgroup 1a and genotype EbpC into subgroup 1d.

Animal hosts of genotype D, EbpC, EbfelA, and L are shown in Table 3. Except for the novel genotype ETMK1, all positive genotypes in the present study have zoonotic potential. A variety of domestic and wild animal hosts have been reported in genotype D and EbpC, while only felines have been reported as an animal host in genotype EbfelA and L.

**Table 3.** Animal hosts in *E. bieneusi* genotype D, EbpC, EbfelA, and L in published records.

Genotype	Subtype	Animal hosts	Country	References
D	1a	Domestic animals		
		Cattle	Korea	[18] [19]
			USA	[31]
		Dog	Portugal	[24]
		Horse	Colombia	[32]
		Pig	Czech Republic	[28]
			Japan	[1]
			USA	[5]
		Wild animals		
		Beaver	USA	[34]
		Falcon	Abu Dhabi	[26]
		Macaque	USA	[34]
			Germany	[11]
		Muskrat	USA	[34]
		Raccoon	USA	[34]
EbpC	1d	Domestic animals		
		Pig	Thailand	[22]
			Japan	[1]
			Germany	[27]
		Wild animals		
		Beaver	USA	[34]
		Fox	USA	[34]
		Muskrat	USA	[34]
		Otter	USA	[34]
EbfelA	1a	Feline	Switzerland	[25]
L	1a	Feline	Germany	[7]

## Discussion

The zoonotic potential of the *E. bieneusi* positive samples in the community and predominance of *E. bieneusi* genotype D are key distinguishing features in this study. In Thailand, the most prevalent genotype in HIV patients was reported to be zoonotic genotype D [22]. However, only anthroponotic genotype A has been identified in the community or non-HIV individuals [20, 21]. This is the first report in Thailand to identify zoonotic genotype D in the community.

Prevalence of intestinal microsporidiosis in HIV patients varies widely from 1.5% to 50%, depending on differences in geographic region and diagnostic method [2]. In developed countries, prevalence of intestinal microsporidiosis has decreased after the propagation of highly active antiretroviral therapy (HAART) [12, 39]. However in developing areas, intestinal microsporidiosis is still highly prevalent among HIV patients due to the limited availability of HAART [13]. In addition, microsporidia have been recently identified in non-HIV immunosuppressed individuals, such as organ-transplant recipients, children, the elderly, and patients with malignancy and diabetes [10].

In this study, the prevalence of *E. bieneusi* infection was 3.8% and 2.9% in Western and Northern Thailand, respectively. Evaluation and comparison of the prevalence with previous studies are difficult due to a paucity of investigations in the community as well as differences in conditions. In Thailand, *E. bieneusi* has been detected in young children in orphanages; the prevalence was 4.1% by microscopy [21]. In communities around pig farms, the prevalence was 1.4% by microscopy [20]. Taking into consideration these previous studies in Thailand, the infection rate in our investigation is within expectations.

*E. bieneusi* genotype is influenced by geography. In European countries, genotype B has been most frequently detected followed by genotypes A and C [3, 7]. These genotypes have been reported in humans only. In Africa, zoonotic genotype K has been frequently identified in Uganda and Gabon [4, 38], while anthroponotic genotype A was the most prevalent in Cameroon and Niger [4, 13]. In Latin America, anthroponotic genotype A was most commonly found, followed by zoonotic genotype Type IV and D in Peru [34]. In Australia, only genotype B has been reported as a causative genotype [33]. In China, CHN1, 3, and 4 were all reported, each of which has potential for zoonotic transmission [40].

Overall, anthroponotic genotypes are commonly seen in developed countries, while both anthroponotic and zoonotic genotypes are observed in developing areas. Opportunities for zoonotic transmission are assumed to be higher in developing countries, especially in rural parts due to frequent animal contact. The transmission routes of *E. bieneusi* are still not completely understood. Several transmission routes, including direct person-to-person, zoonotic, and food and waterborne, have been reported [9]. With regard to zoonotic transmission, several genotypes have been identified from both humans and animals; genotypes with broad host specificity may be responsible for the zoonotic transmission [30]. Additionally, Cama et al. [6] reported possible zoonotic transmission from domestic guinea pigs to a child with no evidence of immunosuppression. In the present study, involvement of zoonotic transmission routes has been observed. Food and waterborne transmission, however, cannot be ruled out since both serve as vehicles for the organism.

Distribution of genotype is reported to be different between immunocompromised and immunocompetent hosts. According to Liguory et al. [23], in France, genotype B was most frequently observed in HIV patients, whereas genotype C in non-HIV-infected patients. Both genotypes have been reported in humans only. Similar results were observed in the Netherlands; genotype C was identified in non-HIV patients only. Genotype D, the most prevalent genotype in this study, is widely distributed, and is often reported in humans in many countries in Europe, Africa, Latin America, and Asia. However, genotype D has always been identified in HIV patients, except for three HIV-negative individuals identified in a rural community in Cameroon [37]. Although the factors influencing these differences in genotype distribution are still undetermined, the involvement of host immunity, pathogenicity of the organism, and routes of transmission have been hypothesized [23].

Genotype D and EbpC have less host specificity. Genotype D has been reported in a wide range of animals: domestic animals such as dogs, horses, and swine, and in wild animals such as beavers, falcons, fox macaques, muskrats, and raccoons [30]. Genotype EbpC also has been identified in a broad range of wild animal hosts, but not in domestic animals; only in pigs has it been reported. It is assumed that transmission between humans, domestic and wild animals occurs in these genotypes. In Thailand, genotype EbpC has frequently been identified in pigs, and they may be the source of transmission in the area [20]. ETMK1, a novel genotype in this study, was one base different from genotype L, V, and EbfelA. Genotype L and EbfelA are as yet reported in felines only [30], however, genotype V has been identified in humans. Considering the similarity, ETMK1 may have zoonotic potential especially related with felines.

Analysis of the genetic proximity of the *E. bieneusi* ITS sequences from our study with those previously published in genetic databases demonstrated that genotype D and ETMK1 were classified into subgroup 1a and genotype EbpC into subgroup 1d. According to Thellier and Breton [37], the largest groups (Group 1) consist of both anthroponotic and zoonotic strains, whereas the other groups consist of host-adapted zoonotic strains with low public health priority. Group 1 is divided into eight major subgroups: subgroup 1a and 1d are large, both human or animal-specific and human-animal common genotypes are classified into the clades. There is a problem in constructing a phylogenetic tree in *E. bieneusi* ITS sequence – it is the only available polymorphic marker in *E. bieneusi* and is not reliable enough for statistical support. Detailed subtype classification therefore differs among researchers, resulting in confusion in the classification itself, and hampering evaluation of host specificity of the organism [4, 11, 17, 37]. New sets of markers will be required for further analysis [29].

This study had some limitations. First, host immunity such as HIV status was not investigated in the present study; immunocompromised patients may have a higher infection rate of *E. bieneusi* zoonotic strains. Second, the sample size was not large enough to fully analyze genotype characteristics in the community. Third, only human samples were collected in this study; animal samples are required to evaluate further zoonotic transmission routes. The present study is a preliminary study and we are planning a large-scale longitudinal study in which animal and environmental samples, in addition to human samples, will be collected and investigated comprehensively.

In conclusion, this investigation demonstrated zoonotic strains of *E. bieneusi* with a predominance of genotype D in rural communities in Thailand. Our findings show possible zoonotic transmission of *E. bieneusi* in rural communities in Western and Northern Thailand. A future large-scale study to investigate humans and animals, as well as the improvement of available phylogenetic tools, will be required to elucidate epidemiological characteristics of *E. bieneusi*.

## References

[1] Abe N, Kimata I. 2010 Molecular survey of *Enterocytozoon bieneusi* in a Japanese porcine population. Vector Borne and Zoonotic Diseases, 10(4), 425–427 19725762 10.1089/vbz.2009.0039

[2] Anane S, Attouchi H. 2010 Microsporidiosis: epidemiology, clinical data and therapy. Gastroenterologie Clinique et Biologique, 34(8–9), 450–464 20702053 10.1016/j.gcb.2010.07.003

[3] Breitenmoser AC, Mathis A, Burgi E, Weber R, Deplazes P. 1999 High prevalence of *Enterocytozoon bieneusi* in swine with four genotypes that differ from those identified in humans. Parasitology, 118(Pt 5), 447–453 10363277 10.1017/s0031182099004229

[4] Breton J, Bart-Delabesse E, Biligui S, Carbone A, Seiller X, Okome-Nkoumou M, Nzamba C, Kombila M, Accoceberry I, Thellier M. 2007 New highly divergent rRNA sequence among biodiverse genotypes of *Enterocytozoon bieneusi* strains isolated from humans in Gabon and Cameroon. Journal of Clinical Microbiology, 45(8), 2580–2589 17537939 10.1128/JCM.02554-06 PMC1951207

[5] Buckholt MA, Lee JH, Tzipori S. 2002 Prevalence of *Enterocytozoon bieneusi* in swine: an 18-month survey at a slaughterhouse in Massachusetts. Applied and Environmental Microbiology, 68(5), 2595–2599 11976142 10.1128/AEM.68.5.2595-2599.2002 PMC127518

[6] Cama VA, Pearson J, Cabrera L, Pacheco L, Gilman R, Meyer S, Ortega Y, Xiao L. 2007 Transmission of *Enterocytozoon bieneusi* between a child and guinea pigs. Journal of Clinical Microbiology, 45(8), 2708–2710 17537930 10.1128/JCM.00725-07 PMC1951230

[7] Dengjel B, Zahler M, Hermanns W, Heinritzi K, Spillmann T, Thomschke A, Löscher T, Gothe R, Rinder H. 2001 Zoonotic potential of *Enterocytozoon bieneusi*. Journal of Clinical Microbiology, 39(12), 4495–4499 11724868 10.1128/JCM.39.12.4495-4499.2001 PMC88572

[8] Desportes I, Le Charpentier Y, Galian A, Bernard F, Cochand-Priollet B, Lavergne A, Ravisse P, Modigliani R. 1985 Occurrence of a new microsporidan: *Enterocytozoon bieneusi* n.g., n. sp., in the enterocytes of a human patient with AIDS. Journal of Protozoology, 32(2), 250–254 4009510 10.1111/j.1550-7408.1985.tb03046.x

[9] Didier ES. 2005 Microsporidiosis: an emerging and opportunistic infection in humans and animals. Acta Tropica, 94(1), 61–76 15777637 10.1016/j.actatropica.2005.01.010

[10] Didier ES, Weiss LM. 2011 Microsporidiosis: not just in AIDS patients. Current Opinion in Infectious Diseases, 24(5), 490–495 21844802 10.1097/QCO.0b013e32834aa152 PMC3416021

[11] Drosten C, Laabs J, Kuhn EM, Schottelius J. 2005 Interspecies transmission of *Enterocytozoon bieneusi* supported by observations in laboratory animals and phylogeny. Medical Microbiology and Immunology, 194(4), 207–209 15864680 10.1007/s00430-005-0240-y

[12] Dworkin MS, Buskin SE, Davidson AJ, Cohn DL, Morse A, Inungu J, Adams MR, McCombs SB, Jones JL, Moura H, Visvesvara G, Pieniazek NJ, Navin TR. 2007 Prevalence of intestinal microsporidiosis in human immunodeficiency virus-infected patients with diarrhea in major United States cities. Revista do Instituto de Medicina Tropical de Sao Paulo, 49(6), 339–342 18157397 10.1590/s0036-46652007000600001

[13] Espern A, Morio F, Miegeville M, Illa H, Abdoulaye M, Meyssonnier V, Adehossi E, Lejeune A, Cam PD, Besse B, Gay-Andrieu F. 2007 Molecular study of microsporidiosis due to *Enterocytozoon bieneusi* and *Encephalitozoon intestinalis* among human immunodeficiency virus-infected patients from two geographical areas: Niamey, Niger, and Hanoi. Vietnam. Journal of Clinical Microbiology, 45(9), 2999–3002 10.1128/JCM.00684-07 PMC2045311 17634305

[14] Fedorko DP, Nelson NA, Cartwright CP. 1995 Identification of microsporidia in stool specimens by using PCR and restriction endonucleases. Journal of Clinical Microbiology, 33(7), 1739–1741 7665639 10.1128/jcm.33.7.1739-1741.1995 PMC228260

[15] Franzen C, Muller A. 1999 Molecular techniques for detection, species differentiation, and phylogenetic analysis of microsporidia. Clinical Microbiology Reviews, 12(2), 243–285 10194459 10.1128/cmr.12.2.243 PMC88917

[16] Garcia LS. 2002 Laboratory identification of the microsporidia. Journal of Clinical Microbiology, 40(6), 1892–1901 12037040 10.1128/JCM.40.6.1892-1901.2002 PMC130667

[17] Henriques-Gil N, Haro M, Izquierdo F, Fenoy S, del Aguila C. 2010 Phylogenetic approach to the variability of the microsporidian *Enterocytozoon bieneusi* and its implications for inter- and intrahost transmission. Applied and Environmental Microbiology, 76(10), 3333–3342 20228101 10.1128/AEM.03026-09 PMC2869117

[18] Lee JH. 2007 Prevalence and molecular characteristics of *Enterocytozoon bieneusi* in cattle in Korea. Parasitology Research, 101(2), 391–396 17323141 10.1007/s00436-007-0468-0

[19] Lee JH. 2008 Molecular detection of *Enterocytozoon bieneusi* and identification of a potentially human-pathogenic genotype in milk. Applied and Environmental Microbiology, 74(5), 1664–1666 18192409 10.1128/AEM.02110-07 PMC2258614

[20] Leelayoova S, Piyaraj P, Subrungruang I, Pagornrat W, Naaglor T, Phumklan S, Taamasri P, Suwanasri J, Mungthin M, et al. 2009 Genotypic characterization of *Enterocytozoon bieneusi* in specimens from pigs and humans in a pig farm community in Central Thailand. Journal of Clinical Microbiology, 47(5), 1572–1574 19321724 10.1128/JCM.00187-09 PMC2681837

[21] Leelayoova S, Subrungruang I, Rangsin R, Chavalitshewinkoon-Petmitr P, Worapong J, Naaglor T, Mungthin M. 2005 Transmission of *Enterocytozoon bieneusi* genotype a in a Thai orphanage. American Journal of Tropical Medicine and Hygiene, 73(1), 104–107 16014843

[22] Leelayoova S, Subrungruang I, Suputtamongkol Y, Worapong J, Petmitr PC, Mungthin M. 2006 Identification of genotypes of *Enterocytozoon bieneusi* from stool samples from human immunodeficiency virus-infected patients in Thailand. Journal of Clinical Microbiology, 44(8), 3001–3004 16891527 10.1128/JCM.00945-06 PMC1594660

[23] Liguory O, Sarfati C, Derouin F, Molina JM. 2001 Evidence of different *Enterocytozoon bieneusi* genotypes in patients with and without human immunodeficiency virus infection. Journal of Clinical Microbiology, 39(7), 2672–2674 11427592 10.1128/JCM.39.7.2672-2674.2001 PMC88208

[24] Lobo ML, Xiao L, Cama V, Stevens T, Antunes F, Matos O. 2006 Genotypes of *Enterocytozoon bieneusi* in mammals in Portugal. Journal of Eukaryotic Microbiology, 53(Suppl 1), S61–S64 17169069 10.1111/j.1550-7408.2006.00174.x

[25] Mathis A, Breitenmoser AC, Deplazes P. 1999 Detection of new *Enterocytozoon* genotypes in faecal samples of farm dogs and a cat. Parasite, 6(2), 189–193 10416194 10.1051/parasite/1999062189

[26] Muller MG, Kinne J, Schuster RK, Walochnik J. 2008 Outbreak of microsporidiosis caused by *Enterocytozoon bieneusi* in falcons. Veterinary Parasitology, 152(1–2), 67–78 18166273 10.1016/j.vetpar.2007.11.019

[27] Reetz J, Nockler K, Reckinger S, Vargas MM, Weiske W, Broglia A. 2009 Identification of *Encephalitozoon cuniculi* genotype III and two novel genotypes of *Enterocytozoon bieneusi* in swine. Parasitology International, 58(3), 285–292 19318131 10.1016/j.parint.2009.03.002

[28] Sak B, Kvac M, Hanzlikova D, Cama V. 2008 First report of *Enterocytozoon bieneusi* infection on a pig farm in the Czech Republic. Veterinary Parasitology, 153(3–4), 220–224 18342450 10.1016/j.vetpar.2008.01.043

[29] Santin M, Fayer R. 2009 *Enterocytozoon bieneusi* genotype nomenclature based on the internal transcribed spacer sequence: a consensus. Journal of Eukaryotic Microbiology, 56(1), 34–38 19335772 10.1111/j.1550-7408.2008.00380.x

[30] Santin M, Fayer R. 2011 Microsporidiosis: *Enterocytozoon bieneusi* in domesticated and wild animals. Research in Veterinary Science, 90(3), 363–371 20699192 10.1016/j.rvsc.2010.07.014

[31] Santin M, Trout JM, Fayer R. 2005 *Enterocytozoon bieneusi* genotypes in dairy cattle in the eastern United States. Parasitology Research, 97(6), 535–538 16167161 10.1007/s00436-005-1482-8

[32] Santin M, Vecino JA, Fayer R. 2010 A zoonotic genotype of *Enterocytozoon bieneusi* in horses. Journal of Parasitology, 96(1), 157–161 19799490 10.1645/GE-2184.1

[33] Stark D, van Hal S, Barratt J, Ellis J, Marriott D, Harkness J. 2009 Limited genetic diversity among genotypes of *Enterocytozoon bieneusi* strains isolated from HIV-infected patients from Sydney, Australia. Journal of Medical Microbiology, 58(Pt 3), 355–357 19208886 10.1099/jmm.0.006445-0

[34] Sulaiman IM, Fayer R, Lal AA, Trout JM, Schaefer FW, 3rd, Xiao L. 2003 Molecular characterization of microsporidia indicates that wild mammals Harbor host-adapted *Enterocytozoon* spp. as well as human-pathogenic *Enterocytozoon bieneusi*. Applied and Environmental Microbiology, 69(8), 4495–4501 12902234 10.1128/AEM.69.8.4495-4501.2003 PMC169096

[35] Tamura K, Dudley J, Nei M, Kumar S. 2007 MEGA4: Molecular Evolutionary Genetics Analysis (MEGA) software version 4.0. Molecular Biology and Evolution, 24(8), 1596–1599 17488738 10.1093/molbev/msm092

[36] ten Hove RJ, Van Lieshout L, Beadsworth MB, Perez MA, Spee K, Claas EC, Verweij JJ. 2009 Characterization of genotypes of *Enterocytozoon bieneusi* in immunosuppressed and immunocompetent patient groups. Journal of Eukaryotic Microbiology, 56(4), 388–393 19602086 10.1111/j.1550-7408.2009.00393.x

[37] Thellier M, Breton J. 2008 *Enterocytozoon bieneusi* in human and animals, focus on laboratory identification and molecular epidemiology. Parasite, 15(3), 349–358 18814706 10.1051/parasite/2008153349

[38] Tumwine JK, Kekitiinwa A, Nabukeera N, Akiyoshi DE, Buckholt MA, Tzipori S. 2002 *Enterocytozoon bieneusi* among children with diarrhea attending Mulago Hospital in Uganda. American Journal of Tropical Medicine and Hygiene, 67(3), 299–303 12408671 10.4269/ajtmh.2002.67.299

[39] van Hal SJ, Muthiah K, Matthews G, Harkness J, Stark D, Cooper D, Marriott D. 2007 Declining incidence of intestinal microsporidiosis and reduction in AIDS-related mortality following introduction of HAART in Sydney, Australia. Transactions of the Royal Society of Tropical Medicine and Hygiene, 101(11), 1096–1100 17662322 10.1016/j.trstmh.2007.06.003

[40] Zhang X, Wang Z, Su Y, Liang X, Sun X, Peng S, Lu H, Jiang N, Yin J, Xiang M, Chen Q. 2011 Identification and genotyping of *Enterocytozoon bieneusi* in China. Journal of Clinical Microbiology, 49(5), 2006–2008 21389159 10.1128/JCM.00372-11 PMC3122652

